# The potential of facial nevi in personal identification

**DOI:** 10.1038/s41598-024-56847-z

**Published:** 2024-03-14

**Authors:** Annalisa Cappella, Riccardo Solazzo, Debora Mazzarelli, Daniele Gibelli, Claudia Dolci, Chiarella Sforza, Cristina Cattaneo

**Affiliations:** 1 Dipartimento di Scienze Biomediche per la Salute, Università degli Studi di Milano, 20133 Milan, Italy; 2 U.O. Laboratorio di Morfologia Umana Applicata, IRCCS Policlinico San Donato, 20097 San Donato Milanese, Italy; 3 LAFAS (Laboratorio di Anatomia Funzionale dell’Apparato Stomatognatico), Dipartimento di Scienze Biomediche per la Salute, Università degli Studi di Milano, 20133 Milan, Italy; 4 LABANOF (Laboratorio di Antropologia e Odontologia Forense), Sezione di Medicina Legale, Dipartimento di Scienze Biomediche per la Salute, Università degli Studi di Milano, 20133 Milan, Italy

**Keywords:** Translational research, Anatomy, Medical ethics, Three-dimensional imaging

## Abstract

Forensic anthropologists dealing with personal identification (PI) of human remains have recently stressed the need to explore the potential of “secondary identifiers” for identifying victims who died in particular events for whom images often represent the main antemortem data available. Being the face the part most exposed in images, characteristics as pigmented skin lesions (PSLs), can be crucial if combined with other input. Since no data is available on frequencies and distribution of facial PSLs in the general population, this study aims at systematically collecting such data to verify their potential in PI and to open a debate on the aid that “secondary identifiers”, regardless of their specific nature, can give to the identification of the deceased in specific forensic contexts. A retrospective analysis on three-dimensional facial models of 1039 Italian subjects (from 4 to 84 years old) was conducted to examine the incidence of PSLs discriminated according to size and position in well-defined facial areas. From the collected data we developed a probabilistic approach providing the likelihood ratio (LR) for two settings: (1) the relative frequencies of nevi in the various facial areas, providing the deriving compound probability of owning a certain facial PSLs pattern; and (2) codes describing the facial nevi pattern of each individual of our population, thus testing their uniqueness and so their potential in PI. The calculated LRs mostly proved high identifying strength, particularly when provided by the compound probability-based approach. Data on incidence and position of facial nevi, their generated codes, and the probabilistic approach here presented, all constitute a starting point for advancing secondary identifiers. Nonetheless, although this preliminary study proved facial PSLs as valuable and potentially useful for identification, their significance and validity should be interpreted with caution as we are still at the first theoretical step clearly based on ideal conditions, and thus further investigations are due on the limitations of their use in practical identifying settings. Therefore, being this systematic study only a preliminary one in its nature, it is recommended not to use this kind of approach until further studies will test its validity in several practical conditions.

## Introduction

Forensic anthropology has recently experienced a vivid progress and, since its beginning, this discipline has expanded its scopes by developing new scientific knowledge and methods. Nowadays, its current practices are no longer limited to the mere recovery and morphometric analysis of remains but go well beyond^1–3^. Forensic anthropology applications might concern both dead and living persons and they cover many aspects: from the biological profiling and personal identification procedures^4–7^ to the analysis of trauma and traces (just to mention some)^8–12^, thus requiring many scientific approaches^9,11,13^, often multidisciplinary^1^. The advances gained by the field are the response to the many complex and global challenges encountered in caseworks or mass disasters, no matter if of medico-legal or humanitarian nature. To comply with these necessities, forensic anthropologists have been more and more pushed in designing new research to seek solutions and answers, as evidenced by the current vast anthropological literature.

Among the many aspects that have been recently investigated by the researchers of the discipline, one of the ‘hottest’ topics that has lately gained much attention is that of the so-called “secondary identifiers”. Interpol has traditionally divided identifying features into primary (namely DNA, fingerprints and dental evidence) and secondary (osteological features, implants, other personal descriptors and initially personal belongings) identifiers. However, more and more biological secondary identifiers (not personal belongings) are being treated as primary ones. The so-called, mainly in the past, secondary identifiers may be particularly helpful in personal identification^14,15^, especially in the humanitarian contexts concerning migration where, during the desperate journey, many fatalities occur involving even hundreds of victims^16,17^. A striking example is represented by the Lampedusa shipwreck of October 3rd, 2013, where over 366 individuals died, as explained thereafter. In such contexts—due to many factors often linked to the social and economic conditions of the country of origin—the identification procedures based on primary identifiers might be slightly or not applicable, mostly because there is no chance to obtain ante-mortem data to compare such as genetic samples, medical and dental records^18–20^, or because most post-mortem data are unrecoverable due to the poor state of preservation of the bodies^17^. Hence, the board members of the Forensic Anthropology Society of Europe (FASE) have recently called the attention on the “secondary identifiers”, also generically referred to as “forensic anthropological identifiers”, highlighting the urge to verify whether they could be valid alternatives to primary identifiers (when failing), and in that case to build up on them new methods leading to conclusive personal identifications.

In detail, the personal, or positive, identification of an individual is routinely performed by comparing data of the missing person and data of the individual to be identified (respectively ante-mortem (AM) and post-mortem (PM) sets of data in case the individual is dead)^21^. In the identification process, it is auspicial that forensic experts from different fields cooperate in a holistic and exhaustive investigation for confirming or excluding an hypothesis of identity^22^. Indeed, multiple forensic profiling methods rather than a single one are recommended for achieving an identification even when the approaches are based on primary identifiers^18,22–25^.

“Primary identifiers”-based methods, entailing genetics, fingerprint analysis, and odontology, are all along considered as the elective methods that allow to more reliably confirm or exclude an identity, thus leading to a conclusive identification per se. Instead, the “secondary identifiers” could be defined as all those features that, not being primary identifiers, characterize a person although they are not necessarily and unreservedly individualizing since they are potentially shared by multiple persons. Thus, secondary identifiers, if not anatomically and unequivocally defined ‘unique’, should be used in combination given that it would be unlikely that two individuals share the same “characterizing pattern” if multiple identifiers are considered together.

Medical data and related images, photos, descriptions by relatives are all excellent sources for the so-called “personal descriptors” which might concern external features (like tattoos, scars, pigmented skin lesions (PSLs), and facial and ear shapes and features) or internal morphological features (as skeletal ones, medical devices and prosthesis or other body part characteristics). The literature shows many case studies focused on diverse kinds of secondary identifiers with the intent to provide ‘insight’ for the personal identification: ante-mortem fractures^9,26^, orthopaedic or medical devices^27–29^, bone morphology and anatomical features^30–38^, bone degenerative changes^39,40^, skeletal and surgical features^41–44^, tattoos^45–47^, scars^48,49^, melanocytic naevi^50,51^, wrinkles^52^, vessel patterns^53^, trauma^54,55^. In some circumstances, also clothing and personal belongings can be a precious source of information in the identification process but can never have a unique value in identification^56,57^.

Regardless of the type of feature investigated for the purpose of personal identification—whether concerning skeletal, soft tissues, or other body part features—these are only some of the examples surveyed to explore the potential of secondary identifiers proving the increasing interest of anthropologists who are among the experts called to pursue such analyses^15^. Indeed, they are well aware that any detail, sometimes even the smallest one, whether directly detected on the remains or indirectly on photos, videos, radiological images or whichever material, becomes an opportunity to confirm or exclude the hypothesis of identity of a person. Of course, secondary identifiers have not been as deeply investigated as the primary ones, and only few systematic studies have investigated their potential identification strength also determining their probative value, hence their “uniqueness”^38,41,42,50,58^. In this perspective, Blau et al.^14^ recently stated that, in order to reach a conclusive identification, it is not important whether a feature is “unique” in the literal meaning of the term, but rather whether the analysed traits are “unique” enough to be those of the person of interest in the analysed context, a concept also known as “Parent Population”^59^, “Identification Universe”^60^, or “Population at large”^61^. Thus, a trait may be of null significance in the context of the entire global population because it is possessed by numerous individuals, but in the context of the analysed framework it can be potentially possessed by one individual only: the personal identification can be based on whichever trait that can prove the identity of an individual beyond any reasonable doubt^14,15,17^ in a specific scenario.

Besides, the usefulness of a means of identification depends on the analysed context and it is not possible to know a priori which type of information will lead to a positive identification: a reliable identification can be potentially achieved via any pattern and be possible in any context once the analysed feature shows a sufficiently large variability among the population^53^ once tested and described. In fact, as stressed by Grivas and Komar^62^, forensic anthropologists are more and more requested in scientific frameworks and courtrooms to quantify the value and weight of evidence under the standards established by Daubert^63^ or Kumho^64^. In other words, morphological and anatomical features used for the personal identification, either in living persons suspected of a specific crime or dead persons for whom a suspect of identity exists, will become to all effect evidence of identity once their frequency in the general population is verified and their probative value as potential identifiers systematically assessed.

To these purposes, as reported in a recent study^65^, facial characteristics with a potential ‘individualizing significance’ can be often easily detectable in photos provided from relatives searching their missing loved ones, and sometimes even noticeable in digital images posted on their social networks^66^, which are widely diffused nowadays^67,68^. Particularly, facial nevi and similar marks, clinically called pigmented skin lesions (PSLs), can have a significant potential in the identification process of those contexts where photos or videos (provided by relatives or found on social networks accounts) result the only source of AM data, as occurred for the identification of the migrants died in the Lampedusa shipwreck of October 3rd, 2013, occurred in the Mediterranean Sea^19,65^. In this context, genetic and fingerprinting analyses were inapplicable or inconclusive for the positive identification of several victims: in these cases, experts turned to forensic anthropological identifiers as teeth, ears, moles, scars, and tattoos that, although considered non-traditional primary identifiers, were detectable on the AM images, demonstrating to be more relevant in such a context^19^. Particularly facial nevi were found to be numerous in many victims and so were used for reinforcing the conclusive identification hypothesis, fostering the need to further explore their potential in personal identification. Overall, the high incidence of nevi in a population is reported mainly in epidemiological and dermatological studies and its trend peaks in the second decade of life, then mostly stabilises in the following decades until reaching the 7^th^ decade when it follows an inconstant trend (either increases or disappears)^69–74^. However, the data reported in literature are limited and generic: no systematic data specifically on the frequency of PSL in the face is available, neither from epidemiological research nor from forensic studies. In forensics, the use of pigmented skin lesions in identification is not a new approach and their potential has been already investigated in hands^50,51^. With the intent to solve a case of video identification of a man suspected of indecent assault on a child, Black et al.^50^ investigated hand melanocytic nevi in terms of incidence and position on the dorsum of the hands, specifically in “cells” determined by landmarks, finding that nevi can really represent valid proof in court^50^. In fact, the authors conducted a systematic study on hands of a large population whose profile proxied the one of the suspect in terms of sex and age. The incidence of hand nevi in specific hand cells was verified and so was their evidential weight based on likelihood ratios. Through the comparison between hand images of the reference population and those of the suspect, the likelihood ratio was calculated and thus used to confirm the identity of the suspect in court. Its evaluation resulted as of moderate support when interpreted according to the standards suggested by the Association of Forensic Science Providers (AFSP)^75^. Later, Malone^51^ assessed the frequency and distribution of nevi and scars in 14 segmented regions of the dorsum of hands in a Canadian population. This second study analysed only the frequency of pigmented skin lesions in hands, thus making a descriptive analysis, but without attempting any statistical approach.

To the best of our knowledge, no systematic studies investigating the frequency of pigmented skin lesions (PSLs) in the face have been conducted so far with a similar intent.

Our study is built upon the methodological, procedural and statistical approaches used by the mentioned studies; hence, the aim of the present study is twofold:

1. To systematically survey the frequency of facial pigmented skin lesions in a large reference population (more than 1000 individuals) verifying possible differences among individuals of diverse sex and age,
2. To evaluate whether such facial descriptors might assume peculiar and identifying descriptive patterns in different individuals, so quantifying their strength of evidence in identification through two different statistical approaches: compound frequency probability and related likelihood ratio (LR), and likelihood ratio concerning facial patterns described by “descriptive” codes.

We are not proposing a new method of identification based on facial nevi, but rather we are highlighting the potential that such characteristics might gain in challenging scenarios requiring a holistic identifying approach. Indeed, likelihood ratio is a statistical means for presenting the strength of evidence widely accepted in the forensic discipline: thus, it is reasonable to believe that it is also relevant for exploring the potential of facial nevi for personal identification.

## Materials and methods

This retrospective study was conducted on three-dimensional (3D) facial models of individuals of both sexes with an age ranging from 4 to 84 years. The 3D models were obtained through the Vectra^®^ M3 3D Imaging System (Canfield Scientific, Parsippany, NJ, USA), which is a safe, non-invasive, fast, and accurate surface imaging optical system^76,77^.

The analysed three-dimensional models were selected from a database of faces of more than 2500 individuals. The facial models were included in the study if of good quality and complete, while the presence of beard, moustaches, make-up, and widespread freckles were all criteria of exclusion in order to set up an ideal sample where to verify the accurate data on frequencies and distributions of facial nevi. 3D digital facial models of 1039 different individuals (517 males and 522 females) were thus selected and included in the study sample. The latter was arbitrarily divided in different age-groups in order to test possible differences among different ages: 4–12 years old (childhood), 13–20 years old (adolescence), 21–30 years old (young adulthood), 31–45 years old (middle adulthood), 46–60 years old (late adulthood), and individuals older than 61 years (old age).

### Ethical statement

**Figure 1:**
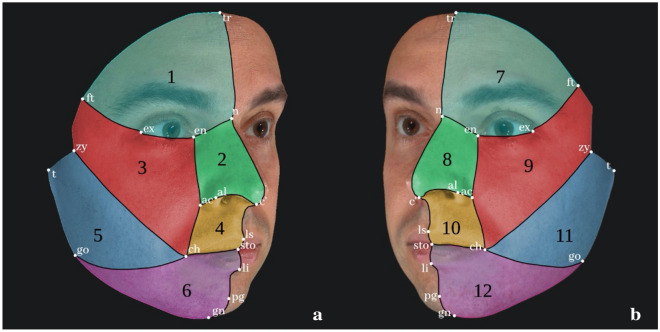
(**a**) Right areas numbered from 1 to 6; (**b**) Left areas numbered from 7 to 12 representative of the subdivision of the face in the 12 facial areas used for counting (and verifying the distribution) of the facial PSLs and for developing the identifying code-system. In the figure the facial areas are separated by lines crossing the anatomical reference landmarks used for their definition: tr: trichion; n: nasion; c’: columella; ls: labiale superius; sto: stomion; li: labiale inferius; pg: pogonion; gn: gnathion; ft: frontotemporale; ex: exocanthion; en; endocanthion; ac: alar crest; al: alare; zy: zygion; t: tragion; ch: cheilion; go: gonion. The bilaterally corresponding areas are coloured alike: 1–7 (light blue); 2–8 (green); 3–9 (red); 4–10 (orange); 5–11 (dark blue); and 6–12 (violet).

**Figure 2:**
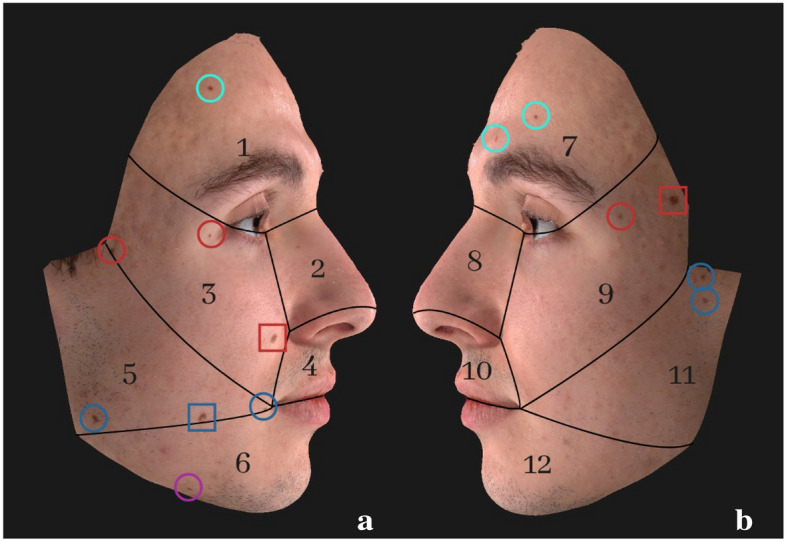
Example of Code I assessment on Subject 9 (a: right side; b: left side). Circles represent nevi with size between 1 and 3 mm, while squares indicate nevi larger than 3 mm in size. Colours represent the facial areas according to those depicted in Fig. 1. Code II can be assessed too, evaluating only the number of nevi per facial area and not considering the size discrimination (square or circle).

This study is part of a greater project on the evaluation of facial morphology which was approved by the local Ethics Committee of the University of Milan (26.03.2014; n° 92/14) and was conducted in accordance with the Declaration of Helsinki^78^. The informed consent was signed by each individual or from their legal tutor/parent in cases of underage individuals. In addition, informed consent to publish images in an online open access publication was obtained from the subjects portrayed in Figs. 1 and 2.

### Observational analysis of three-dimensional facial models

All 1039 selected 3D facial models were investigated for the presence and localization of facial pigmented skin lesions.

In particular, 26 craniofacial landmarks were positioned on the facial surface in order to delineate a facial grid including 12 cells/areas, six for each facial side (Fig. 1). The twelve cells were specifically numbered from 1 to 6 and 7 to 12 for right and left side respectively, going in ascending order from the superior to the inferior part of each facial side, as depicted in Fig. 1. Once each 3D facial model was gridded in the 12 facial cells through the landmarks, an observational analysis based on presence, number, classification by size (1–3 mm or above 3 mm), and location of each facial pigmented skin lesion was conducted and recorded. The use of facial areas anatomically determined by landmarks allowed to verify the distribution of the analysed features (PSLs) in the faces as well as to build the “identifying” code-system.

### Frequencies analysis

Pigmented lesion count included any visible sign (moles and similar) of size over 1 mm that expresses a darker or different colour (black, blue, brown, red, white, pinkish and so on) and clearly defined borders separating it from the surrounding skin. To be included in the count, all pigmented lesions had to be easily detectable when examining the 3D image, thus very faint, indiscernible, or hindered (for instance covered by hair, etc.) signs were not considered, and so excluded from the count.

The simple count and classification of the nevi present within a 3D facial image, and so within each facial region, was performed by a single observer on the entire study sample (1039 individuals). The main observer was an expert forensic anthropologist with more than 6 years of experience in 3D digital imaging and facial analysis. However, the reliability of the protocol used for collecting the data was tested: the intra- and inter-rater reliability was verified by repeating the entire procedure on 120 3D facial models randomly selected from the entire sample.

All the data deriving from the count and analysis conducted on the 1039 3D facial models were recorded in a Excel^79^ dataset on which the second aim of the study, that is the statistical approaches of compound probability and likelihood ratio of facial nevi patterns as explained afterward, was performed.

Relative frequencies of number of nevi per face and per facial area were calculated and subsequently used to verify the compound probability (multiplication of each relative frequency) that one individual would present a certain pattern of traits. In addition, two different codes describing the own facial nevi pattern (explained afterward) were generated for each individual in order to create a dataset as the starting point for the analyses based on the likelihood ratio approach.

Lastly, to test the two statistical approaches, ten individuals not included in the original study sample were analysed, and the compound probability and likelihood ratio of their patterns of features were calculated according to the reference data we collected prior to the experiment.

### Identifying codes analysis

The data gathered by the observation of each facial model concerning the presence, number of PSLs and their position in the 12 delineated facial cells was used to generate two types of code for each subject. “Code I” is a 24 digits-code formulated arranging the number of PSLs discriminated according to size in each of the 12 facial areas, thus two values were available for each cell (the number of PSLs with a size 1–3 mm, and the number of PSLs with a size ≥ 3 mm, with the latter being always the second digit in each cell). “Code II”, less informative, is a 12-digits code formulated arranging the total number of PSLs in each of the 12 facial cells as serial numbers, disregarding the classification by size. The digits’ order in both codes was from facial cell 1 to facial cell 12 as illustrated in Fig. 1 and described in the previous section. Concerning Code I, the number of PSLs with a size of 1–3 mm was always before that of PSLs with a size ≥ 3 mm for each facial cell. In case the total number of nevi in a single facial area exceeded 9, we used the alphabetic letters from A to Z to substitute double-digit numbers. From the built dataset reporting the two codes descriptive for each of the 1039 individuals analysed, we retrieved unique and shared codes and their related numbers. This step allowed us to determine the number of individuals having a code not shared by anyone and the number of individuals sharing their code with others, a crucial step for the following calculation of the likelihood ratio of possessing a specific pattern of PSLs, similarly to Black et al.^50^ and Jackson and Black^80^.

### Data analysis

The inter-operator and the intra-operator reliability were assessed through the Intraclass Correlation Coefficient (ICC) which is a measure of the reproducibility and repeatability of the method. ICC values, and the corresponding 95% Confidence Interval (CI), were calculated based on single measurement, absolute agreement, two-way mixed-effects model (ICC 3,1), as reported by Koo et Li^81^. Once the repeatability was tested, further analyses focused on the descriptive and the inferential statistics in order to obtain empirical data about the analysed “personal descriptors”.

The descriptive statistical analysis of the observed features was conducted for the entire sample and for the different subsamples established by age and sex, and it included the calculation of mean, standard deviation (SD), median, minimum, and maximum values of the PSLs observed for the whole face and not for the single facial areas. The empirically obtained data, due to the scarcity of information about the occurrence of pigmented lesions in the face on the general population, were also used to retrieve a prior knowledge about their frequencies, hence the total number of PSLs per face and per each facial region of the entire sample, and the number of individuals that exhibit a certain number of PSLs in each facial area (‘frequency of occurrence’ expressed as percentage) were calculated.

Statistically significant differences about the total number of PSLs among individuals of same age-group but different sex and individuals of diverse age-group according to sex were evaluated through Generalized Linear Mixed Models (GLMMs) and, in case of statistically significant differences for each factor (age and sex), pairwise comparisons were performed and their p-values adjusted using the step-down Bonferroni correction.

The inferential statistical analyses and the intra- and inter-reliability analyses were performed on SPSS (IBM Corporation, IBM SPSS Statistics for Windows, Armonk, NY, Version 28.0), while the descriptive one was performed on Excel^79^. Alpha was set to 0.05 (statistical significance reached for p-value < 0.05).

The compound probability and the related likelihood ratio for the facial nevi patterns, as well as the likelihood ratio for a specific code of the ten individuals analysed ex novo were calculated on Excel^79^. The compound probability of a certain pattern was obtained by multiplication of relative frequencies (Eq. 1), and the related likelihood ratio (likelihood ratio pattern) was calculated as the inverse of the compound probability (Eq. 2).

1 $$C{ompound \;Probability}_{Pattern}=\prod_{i=1}^{m}{P}_{n,{x}_{i}}$$

where P: probability; n: number of PSLs; x: facial area; and m: 24 for code I and 12 for code II.

2 $${Likelihood \;Ratio}_{Pattern}= \frac{1}{{Compound \;Probability}_{Pattern}}$$

In addition, we also calculated the likelihood ratio of having a specific code, that can be calculated as the ratio of the probability of an observation given a proposition is true (H_0_) to the probability of obtaining the same observation given an alternative mutually exclusive proposition is true (H_1_) (Eq. 3):

3 $${Likelihood \;Ratio}_{Code}= \frac{P{(H}_{0})}{P{(H}_{1})}$$

where P: probability, H_0_: null hypothesis and H_1_: alternative mutually exclusive hypothesis.

For our specific intent, H_0_: “The pattern of nevi is that of the person of interest” and H_1_: “The pattern of nevi is that of another unknown person”. Furthermore, to test our method we assumed that P(H_0_) = 1 since the PSLs pattern of an individual is that of the observed individual.

The denominator probability P(H_1_) is retrievable from the “between-sample” variability, and it can be assigned to a value equal to the probability of the code in the population sample, thus shared by other individuals. Equation 3 can be simplified as seen in Eq. (4), that we used for calculating the likelihood ratio code:

4 $${Likelihood \;Ratio}_{Code}= \frac{1}{{P}_{code}}$$

where P_code_ is the probability of the code in the population sample.

The calculation of values of likelihood ratio, its methodological use, and the overall interpretation were conducted analogously to what was performed by Black et al.^50^ in their study. In particular, the values of each likelihood ratio were interpreted in terms of weight of evidence according to the guidelines suggested by the Association of Forensic Science Providers (AFSP)^75^, as summed in Table 1.

**Table 1:**
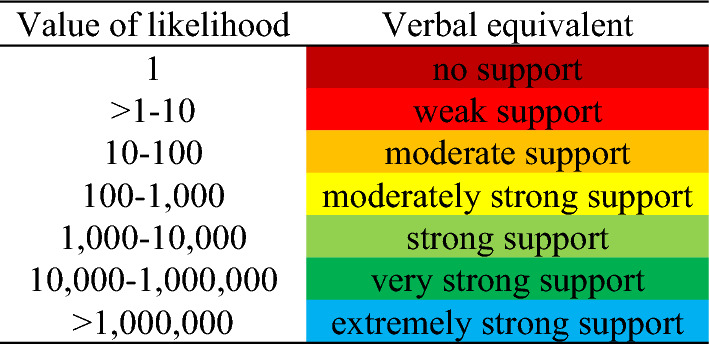
Values of likelihood ratio and related verbal equivalent (in terms of strength).

## Results

The studied sample consisted of 1039 three-dimensional facial models representative of an Italian, thus European population with an age ranging from 4 to 84 years old (mean 33.8 years, SD 18.5 years). The 3D facial models were obtained from individuals of both sexes: the male sample consisted of 517 individuals ranging from 5 to 84 years old (mean 33.1 years, SD 18.1 years), and the female sample included 522 individuals ranging from 4 to 81 years old (mean 34.5 years, SD 18.9 years) subdivided into six different age groups according to the age of the subjects at the time of the 3D acquisition as reported in Table 2.

**Table 2:** Population and subsample classified according to sex and age-group.

Age group (years)	Males (n)	Females (n)	Total (n)
4–12	79	69	148
13–20	81	95	176
21–30	98	85	183
31–45	105	110	215
46–60	115	100	215
≥ 61	39	63	102
Total	517	522	1039

n: number of 3D facial models (equivalent to number of individuals); Total: Sum of the number of individuals.

### Reliability of the method

The Intraclass Correlation Coefficient (ICC) was calculated as a measure to assess the inter-observer reproducibility and intra-observer repeatability of the method, thus its reliability, for the observations concerning the PSLs discriminated according to size (1–3 mm and ≥ 3 mm) and the overall observations disregarding the size (≥ 1 mm). Overall, according to Koo and Li^81^ the method proved an almost excellent or excellent reproducibility and repeatability when the assessment of PSLs was performed by two different operators and by the same operator performing the analysis twice. Hence, the ICC values, and the 95% confidence interval (CI) are all very close to or above 0.900 which is the threshold for the excellent repeatability/reproducibility of the method (Table 3).

**Table 3:** Intra- and inter-observer reliability of the protocol verified by Intraclass Correlation Coefficient and related Confidence intervals.

PSLs size	Intra-observer repeatability		Inter-observer reproducibility	
	ICC value	95% CI	ICC value	95% CI
1–3 mm	0.969	0.966–0.972	0.895	0.885–0.904
≥ 3 mm	0.895	0.886–0.904	0.907	0.898–0.915
All (≥ 1 mm)	0.969	0.966–0.971	0.908	0.900–0.916

ICC: Intraclass Correlation Coefficient; CI: Confidence Interval.

### Descriptive statistics

The mean, standard deviation, median, minimum, and maximum of the number of PSLs for the entire face have been calculated for all subgroups and reported in Table 4. The highest average count of pigmented skin lesions was observed in the individuals belonging to the 13–20- and 21–30-years age groups, for both sexes. Males, except for those included in the 31–45 years age group that have a lower value than age-matched females (5.9 and 6.0 respectively), had a higher average number of pigmented skin lesions when compared to females.

**Table 4:** Descriptive statistics concerning the total number of facial pigmented skin lesions for the whole face according to sex and age groups.

	Age group (years)						
	4–12	13–20	21–30	31–45	46–60	61+	Total
Males							
Mean	4.9	7.9	7.6	5.9	6.9	6.8	6.7
SD	3.6	6.2	6.8	5.6	7.6	7.3	6.4
Median	4	6	6	5	5	3	5
Minimum	0	0	0	0	0	0	0
Maximum	20	30	50	30	44	27	50
Females							
Mean	4.5	6.8	6.0	6.0	4.4	4.1	5.4
SD	3.3	4.6	4.9	5.6	3.3	6.3	4.9
Median	4	6	4.5	4	4	2	4
Minimum	0	0	0	0	0	0	0
Maximum	14	22	25	33	15	33	33

SD: Standard Deviation; Total: Entire population regardless of age-group classification.

The differences in the total number of facial pigmented skin lesions among individuals of different sex but same age-group were evaluated through a GLMM which showed significant differences only for the group of 46–60 years old (p = 0.002), while the following age class (≥ 61 years old) approached the significance (p = 0.054). A slight significance was also obtained in the comparison of the two sexes independently of the age-groups (p = 0.048). The significant differences found between the sexes did not allow to group the whole population together in the following analysis. Thus, the differences concerning the total number of facial pigmented skin lesions for individuals of different age-groups were evaluated independently for the two sexes through a further GLMM analysis. The test revealed statistically significant differences among the diverse age-groups according to the fixed factor ‘sex’ (p < 0.001), thus the pairwise comparisons among the diverse age-groups were performed applying a step-down Bonferroni correction for multiple tests, and the results are reported in Table 5.

**Table 5:**
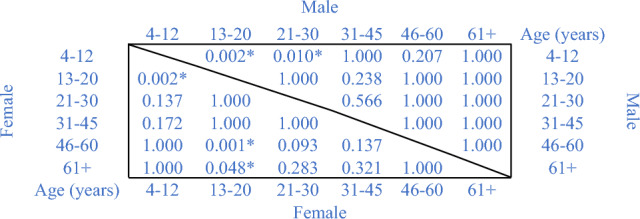
P-values of the pairwise comparison for age in individuals of same sex verified by the GLMM.

*Statistically significant at p < 0.05, adjusted for step-down Bonferroni correction.

### Frequencies analysis

In our reference population only 86 individuals showed no pigmented lesions at all (8.3% of the sample) corresponding to 36 males and 50 females. In particular, the frequency distribution of the total number of facial pigmented lesions was evaluated in both sexes for all age-groups. Most individuals (953 individuals) own at least one pigmented skin lesion in the face (91.7%) and 63.2% of the population has a number of facial features included between 2 and 9, whilst fewer individuals, particularly females, showed a total number of facial pigmented skin lesions ranging between 10 and 15 (12.8%) or greater than 16 (6.0%). Thus, 82% of the population shows at least two lesions in the face. Indeed, the maximum of total number of facial pigmented lesions reached 50 for males and 33 for females. The percentage of individuals having a certain number of facial pigmented lesions in a certain facial area was evaluated as well for the entire sample: while Table 6 summarises data on the total number of nevi observed in the sample, related ranges for face, and percentages of occurrence in each region, Table 7 reports the percentage of individuals with a certain number of pigmented lesions within each region. Most individuals do not express pigmented lesions in facial areas 2 and 4, whilst around 50% of the sample has at least one pigmented skin lesion in facial region 3 or the equivalent region in the opposite side (region 9) with 47% of them (484 individuals) showing more than 2 pigmented skin lesions in the same regions. Thus, the latter is where a pigmented lesion can be more commonly observed. Other facial areas with high frequency of pigmented lesions, even though to a lesser extent than area 3 or 9, are region 1 and the equivalent left region 7, region 5 and the equivalent left region 11, and region 6 and the equivalent left region 12, where the percentage of occurrence ranged between 31 and 39% (percentage of population with at least one pigmented skin lesion in one of these areas).

**Table 6:** Descriptive statistics for number of nevi according to specific facial regions and related frequencies of occurrence.

	Total observed	Range (per face)	Mean (per face)	Standard deviation	Frequency of occurrence (%)
Region 1	757	0–14	0.73	1.31	39.0
Region 2	182	0–5	0.18	0.50	13.2
Region 3	992	0–9	0.96	1.30	51.3
Region 4	153	0–3	0.15	0.39	13.3
Region 5	694	0–9	0.67	1.10	39.7
Region 6	506	0–8	0.49	0.85	33.6
Region 7	658	0–10	0.63	1.17	34.9
Region 8	180	0–5	0.17	0.50	13.8
Region 9	888	0–7	0.86	1.16	48.1
Region 10	130	0–2	0.13	0.36	11.2
Region 11	681	0–9	0.66	1.09	32.6
Region 12	472	0–6	0.45	0.77	31.6
Total	6293	0–50	6.05	5.72	91.7

Total: Entire face without discriminating for regions.

**Table 7:** Percentages of individuals expressing a certain number of nevi in each facial region.

(% of individuals)											
X	0	1	2	3	4	5	6	7	8	9	≥ 10
Region 1	61.0	22.0	9.4	3.7	1.5	1.3	0.4	0.3	0.0	0.2	0.2
Region 2	86.8	9.7	2.9	0.4	0.1	0.1	0.0	0.0	0.0	0.0	0.0
Region 3	48.7	27.1	13.6	6.0	2.5	0.7	0.7	0.4	0.3	0.1	0.0
Region 4	86.7	11.9	1.3	0.1	0.0	0.0	0.0	0.0	0.0	0.0	0.0
Region 5	60.3	24.2	9.1	3.9	1.0	0.8	0.2	0.3	0.1	0.1	0.0
Region 6	66.4	23.4	7.1	2.3	0.3	0.2	0.1	0.1	0.1	0.0	0.0
Region 7	65.1	19.8	8.8	3.5	1.1	0.8	0.5	0.2	0.3	0.0	0.1
Region 8	86.2	11.5	1.5	0.4	0.3	0.1	0.0	0.0	0.0	0.0	0.0
Region 9	51.9	25.1	14.6	4.9	1.8	0.9	0.7	0.1	0.0	0.0	0.0
Region 10	88.8	9.8	1.4	0.0	0.0	0.0	0.0	0.0	0.0	0.0	0.0
Region 11	62.2	21.1	10.7	3.7	1.3	0.4	0.5	0.1	0.1	0.1	0.0
Region 12	68.4	20.9	8.0	2.4	0.2	0.0	0.1	0.0	0.0	0.0	0.0
Total	8.3	9.7	9.9	11.3	9.1	8.5	8.0	6.8	4.4	5.2	18.8

X: Number of pigmented skin lesions; Total: Entire face without discriminating for regions.

### Identifying codes analysis

A total of 830 and 773 “identifying” codes were generated for code I and code II respectively and retrieved from all the total sample (codes from 1039 individuals). Both types of code-systems included the codes representative of those individuals who express no facial pigmented skin lesions having only zeros as digits, and these were shared by 86 individuals. Accordingly, the remaining number of codes, 829 and 772 for code I and II respectively, are representative of the residual 953 individuals having at least one pigmented lesion. Overall, concerning code type I, 783 codes out of the total codes proved unique (94.3%) (as for the example depicted in Fig. 2), while 46 codes describing at least a facial nevus were shared by multiple persons (5.5%) and representative of 170 individuals. Specifically, 19 codes were share by 2 individuals (2.3%), 11 codes shared by 3 individuals (1.3%), and all the rest shared by more than 4 individuals (1.9%).

**Figure 3:**
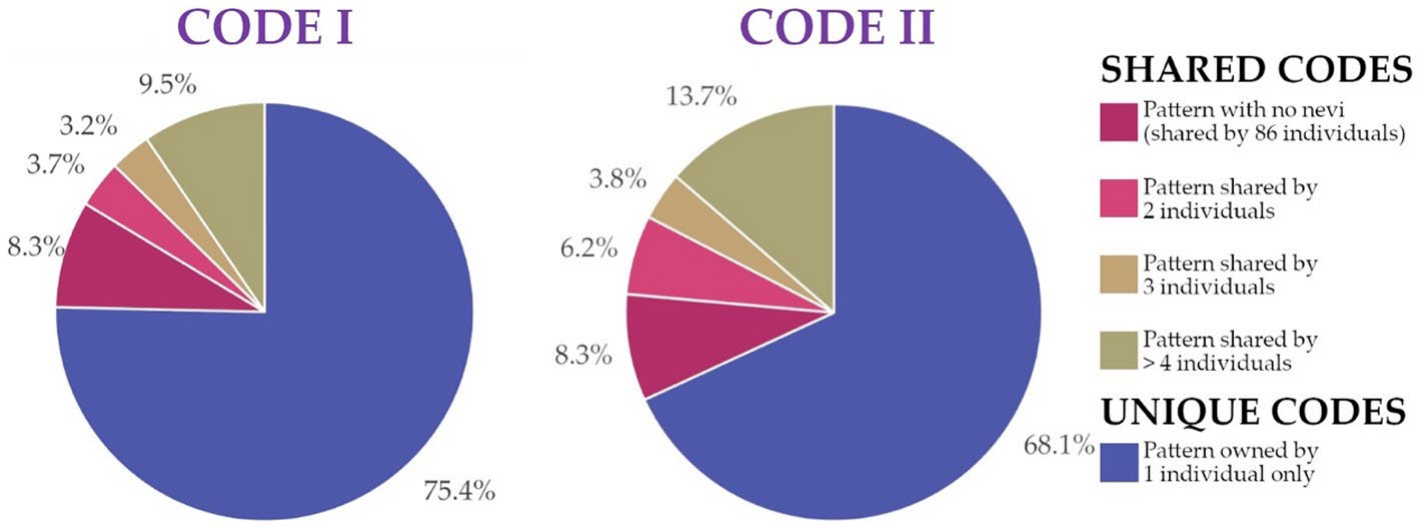
Percentages of individuals with unique and sharing codes (Code I, 24 digits; Code II, 12 digits). Percentage repartition of sharing codes was represented according to number of individuals sharing a specific code (2, 3 or more than 4 persons), including the 86 individuals sharing the code describing lack of nevi (code of zeros).

Concerning code type II, 708 codes out of the total proved univocal (91.6%), and 64 codes descriptive of facial patterns with at least a nevus were shared by more individuals (8.3%) and, specifically, representative of 245 individuals with 32 codes shared by 2 individuals (4.1%), 13 codes shared by 3 individuals (1.7%) and 19 codes shared by more than 4 individuals (2.5%). Data and percentages of individuals expressing univocal or shared codes I and II, including codes descriptive of facial patterns with no nevi, are depicted in Fig. 3.

### Probabilistic approach applied to new examples

Results on the probabilistic approaches (compound probability and likelihood ratio of the pattern and likelihood ratio of the code I) on facial nevi pattern according to size for the ten individuals examined ex novo are reported in Table 8, while related data, which do not consider the size, are reported in Table 9. Strength of evidence goes from moderate to extremely strong support regardless of the size, however, extremely strong support was reached only for likelihood ratio verified for the pattern (compound probability based on frequencies).

**Table 8:**
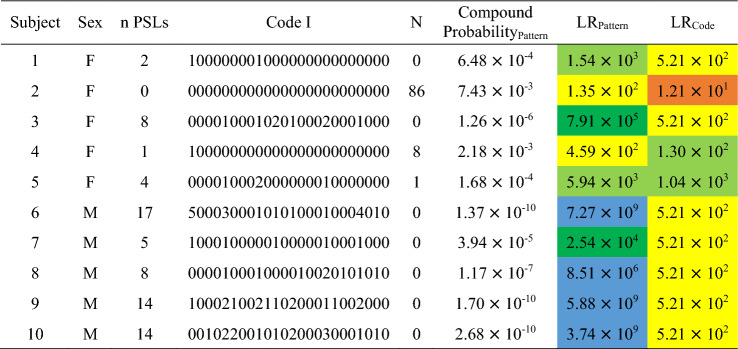
Compound probability of the pattern concerning facial nevi and related likelihood ratio, and likelihood ratio concerning Code I of the ten ex novo individuals tested.

n PSLs: number of facial pigmented skin lesions; N: number of individuals in the database sharing the same code; colors indicate different grades of strength: orange: moderate support; yellow: moderate strong support; light green: strong support; dark green: very strong support; blue: extremely strong support.

**Table 9:**
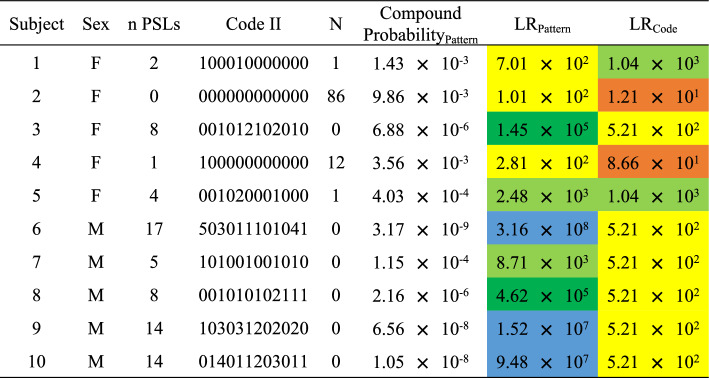
Compound probability of the pattern concerning facial nevi and related likelihood ratio, and likelihood ratio concerning Code II of the ten ex novo individuals tested.

n PSLs: number of facial pigmented skin lesions; N: number of individuals in the database sharing the same code; colours indicate different grades of strength: orange: moderate support; yellow: moderate strong support; light green: strong support; dark green: very strong support; blue: extremely strong support.

## Discussion

Secondary identifiers considered by forensic scientists and anthropologists for aiding personal identification of human remains (regardless the state of preservation) have aroused much attention in the last decades. Recently, they have gained the right exposure to statistical approaches which enable to quantify “the degree of scientific certainty” using likelihood ratios for putative identifications^48,50,61,82,83^, as in the case of DNA-based identification. This approach can be potentially extended to any kind of characteristics, whether skeletal, dental, or physical/skin marks and descriptors. The existence of a plethora of diverse features, once verified as individualizing, can ensure forensic experts to reach identification regardless the kind of available AM information. In fact, if no probative values are examined for “individualizing” descriptors—no matter if ‘primary’ or ‘secondary’—the chances to identify a cadaver through such features with strong evidence become impossible or rather unlikely when the DNA analysis (or fingerprint analysis) is inconclusive or impossible, as in the case of the Lampedusa shipwreck where primary identifiers resulted inappropriate while the secondary ones appeared more pertinent^19^. In this context, because of the kind of the AM material (photos and videos)^65^, although numerous, only facial and body descriptors, that is nevi, scars and tattoos, were found to be possibly used for a comparison in the identification procedure^19^. However, we realized that such descriptors, to be potentially used in identification, must provide probative identification values which have been priorly verified. The latter is a crucial point that has still to be verified for most of these descriptors, including facial nevi, although the urge to address such a gap was reported also in literature^14,15,18,19,22,56,84,85^.

For this reason, our study focused on the analysis of nevi in the face with the intent to verify firstly their frequency in a large population and their distribution in defined facial areas of the face, and secondly the possible existence of unique descriptive facial nevi patterns, thus allowing us to verify if likelihood ratios calculated ex novo for some facial nevi patterns (subjects not included in the analysed sample) could possibly gain a worthy strength value in forensic identification.

Based on our results, we can state three important points: (1) facial pigmented skin lesions are frequent characteristics in the general population analysed in this study, both in terms of presence and numbers regardless of sex and age; (2) detecting facial nevi in diverse facial areas separated according to landmarks is highly repeatable in 3D facial models regardless of the experience of the operator and/or the size of nevi; (3) the statistical framework based on the Bayesian approach used in this study allowed to quantify the probability of possessing a certain facial nevi pattern or to own a certain number of nevi in a specific facial area.

While some individuals lacked pigmented skin lesions in the face (8% of our ‘population at large’), which jeopardizes the use of such descriptor in identification, the high frequency of multiple facial nevi on the remaining population proved their potential. This stresses the discriminating value of this skin descriptor, particularly for those individuals characterized by multiple facial nevi: the higher the number of facial nevi, the more powerful the probative value provided, and the less likely the person shares a certain descriptive code, as suggested from the percentages of unique codes and from findings we obtained once the likelihood ratio was calculated to new faces.

The use of nevi for personal identification is not a novel concept, even though the few works present in the literature assessed these features on hands and not in the face. Black et al.^50^, in 2014, verified the frequency of pigmented skin lesions detected in hands for identification purposes. The authors found much lower frequencies than those we found in faces which, on contrary, has revealed at least one pigmented skin lesion in more than 90% of our population. Therefore, differences in findings between the two studies are evident and likely due to the diverse body region and population analysed, preventing any direct comparison. A similar analysis on hands was conducted by Malone^51^ in a Canadian population in which, similarly to our study, about 91% of the analysed hands exhibited at least one pigmented lesion. Of course, the different findings of the studies, including ours, pose the question concerning diversity among populations and ancestry: frequencies of such features respond not only to genetics, but also to the geographical area where one lives, i.e. exposure to different degrees of ultraviolet radiations and the related behaviours for preventing it. This is to say that, analogously to genetics, specific frequencies of “secondary characters” should be calculated in the various populations, to provide compound probability and likelihood ratios more accurate for the specific population under examination.

Moving to the second point stated above, we can suggest that the detection of nevi in 3D facial models is highly reproducible, no matter the size of nevi, the facial area or the experience of the operator: their assessment always reached excellent or very good repeatability. However, a premise is due: we have verified our frequencies and so reproducibility in 3D stereophotogrammetric facial models which present several advantages instead not present in 2D photos. In fact, the software used for analysing the 3D model allows to magnify images without losing the great resolution, to rotate the face in any direction and to measure very small structures as nevi also smaller than 1 mm: it represents to all effects the ideal situation for properly detecting such descriptors. The same cannot be said for real contexts for which one cannot exclude the presence of pictures of low resolution, or few photos with similar frames, and the difficulty of discriminating the size of pigmented skin lesions, all aspects to be considered^65^. However, this ideal study has been set for calculating the frequencies of such descriptors as the starting point towards their possible use in forensic settings. Further studies should be implemented to verify a similar assessment in 2D images and in real contexts, so clarifying the real practical chance to use such descriptors in identification. However, the two diverse probabilistic approaches were used to specifically overcome this limitation: indeed, the compound probability approach, which generates from relative frequencies for single facial areas, allows to calculate probabilities and likelihood ratio also in those cases where not all facial areas are visible. Of course, in the latter situation the strategy of descriptive codes would be inapplicable, and the likelihood ratio based on the compound approach only partial. In addition, we provided results also in case the discrimination of nevi according to their size is not possible, trying to overcome also this practical limitation.

Coming now to the third point, the performed probabilistic approaches highlighted promising results, paving the way for analogous research on other “secondary identifiers”. The need to use probative values in forensic anthropology was auspicated in the past by several authors^86–90^, not only for identification purposes but in general for any kind of anthropological analysis.

The calculation of the likelihood ratio developed for the two diverse frameworks, one based on the compound probability (thus the relative frequencies) and the other relying on the observed describing codes, emphasizes how statistics can provide highly diversified results starting from the same data. Statistics is a tool for researchers, and it must be consciously used to answer the research questions. The two calculations allowed us to answer different questions and, most importantly, they can be used in different settings. While the likelihood ratio based on the descriptive codes can analyse the number of unique codes in a reference population (percentage of individuals with a unique pattern over the entire ‘population at large’), the statistics based on the compound frequency (pattern) gave instead insight on the probability of a specific pattern of facial PSLs and its relative evidence value in terms of likelihood ratio. The likelihood ratio based on the pattern demonstrated better results than that of the code thanks to the mathematical basis of calculations and, as expected, the likelihood ratios of Code I outperformed that of Code II, providing higher evidential values.

The likelihood ratios calculated ex novo demonstrated similar or even better results than those reported by Black et al.^50^ mostly due to the greater sample size used in our study and the higher frequency of PSLs on the face rather than on the hands. Similarly, our ex novo likelihood ratios do not differ much from some of those concerning DNA presented by Bertoglio et al.^91^, who interpreted likelihood ratios ≥ 10^5^ as sufficient to prove the kinship of two related individuals, and thus to prove the identity of the individual under examination. The study primarily focused on the analysis of 16 autosomal STRs but, whenever the analysis did not allow to reach the willing threshold, the authors increased the number of autosomal loci analysed, and they also extended the analysis to the chromosome-Y loci and the mitochondrial genome. In the same way, the likelihood ratio values of facial nevi patterns could increase if combined with other “secondary” features as scars, tattoos, or unique morphological traits. Furthermore, beside numbers, location and the size of PSLs, these personal descriptors already possess intrinsic peculiar characterizing/identifying features as morphology and shape per se, contours, and colours, which if taken into account could increase the likelihood ratio and the chance to achieve identification, as already demonstrated in previous studies^19^ and for other anatomical structures (i.e. frontal and sphenoid sinuses, bones and bone calluses morphology) ^30,33,35,39,42^. The concept of unique morphological characteristics, in fact, should be keep and used in practices as it is fundamental in the identification process.

Another key aspect to consider is the “identification universe”, particularly in closed mass disasters, which stresses that the obtained insights in terms of evidence findings should be resized on the limited number of analysed individuals rather than on the entire population. This approach may help forensic practitioners in interpreting the results, even those that may not seem particularly promising. However, the knowledge of the value of an identifying descriptor in the general population allows to better interpret and furnish results in all other cases than closed ones, and, in these terms, systematic studies help researchers to understand the potential as well as the predictive values of such features.

### Limitations and future directions

As we have already seen the many advantages that such descriptors might offer to forensic anthropologists in personal identification, we are however not exempt from stating the limits of the present study and of the possible practical use of such descriptors.

The first and main limit of this study stands on the kind of experimental setting: this is an ideal framework, of course auspicial but unlikely to find when working in real practical settings. 3D pictures helped us to build our internal database from which to calculate the frequencies and so the likelihood ratios, but in real contexts the pictures, which one can only hope to be of high quality, will be the material on which to work. The issues deriving from imaging conditions can likely arise as soon as the same survey is performed in a realistic context, and this is a very critical aspect highlighting the preliminary nature of this study, which at this time remains only a first theoretical step and not a practical approach. Indeed, as stated by some authors, in practical unideal conditions morphological comparison by imagery analysis is usually sensitive to loss of quality often caused from blurring, reduction in spatial resolution, angle of incidence, limited perspectives, and lens distortion. If all these factors reduce already the visibility of gross facial features/areas like specific shape of the eyes, mouth and nose^92,93^, one can imagine what will be that of fine details like moles and scars, whose visibility might be even null^65^. Indeed, facial images, whether 3D or 2D, are not always optimal for an identifying comparison regardless the kind of features used for this intent: and nevi are among those details that results difficult to detect in images with low technical qualities^65^. This is the critical point to not underrate, which needs an in-depth pondering when working with imagery analysis. Therefore, future studies analysing images of faces acquired by cameras at different technical and environmental conditions would help in understanding the practical potential of facial nevi detection in real identifying circumstances since it is imperative to recognize the limiting factors that can occur during the image capture^65,92^. Technical factors to consider as limiting ones preventing the detection of fine details like nevi are the image resolution, lens distortions and image compression, while environmental limiting factors might consist in variations of lighting, the pose of the face, the presence of dirty in the lens or the presence of object concealing their visibility, just to mention some. All these limiting factors contribute to reduce the accuracy to detect features useful in the facial analysis for identification purposes as they influence the quality of the image^94^. With these concerns and limitations clearly stated, we auspicate that researchers might take them as investigative opportunities: future studies could address the detection of nevi in unideal practical settings and real circumstances, thus providing unequivocal insights on whether or not they are detectable in practice, and revealing if the approach here presented can be applied or not to real procedures of identification in cadavers. Also, as we focused only on 3D images, which represent the optimal standardized material where to conduct a similar systematic survey and collect the data on the frequencies we found, further studies mainly focused on 2D images are mandatory, as the latter will likely be (regardless their quality) the kind of material more often available in forensic practices for identification purposes^65,91^. Given this important limitation, although most of the facial nevi patterns in our study population proved uniqueness, we still suggest caution in interpreting them as potential identifiers, and await their practical application until further data will be provided by the further validation studies we are trying to encouraging.

The second important limit of the present study to state is the ‘population at large’ used as the reference sample on which to assess the frequencies and from which we have developed the putative strength of nevi in identification. In fact, our ‘population at large’ is only representative of a European population, and specifically an Italian one. Further studies on diverse populations (from a geographically and ethnicity point of view) should be conducted to postulate an overall picture of such facial descriptors. However, the selection of reference sample represents per se one of the most critical aspects in this kind of approaches^61^, especially when findings are applied to identification or to anthropological analyses for profiling remains.

Another concern is the stability of moles: not being a longitudinal analysis, our study only verified the differences in number of nevi among individuals of diverse age classes. The literature still presents few data about their stability over time: their disappearance, changes in colour or size, surgical removals, or even their increase in number, are all aspects to carefully consider during the identification procedure. The few data reported by the literature indicate that pigmented skin lesions (PSLs) are usually present but scarce during childhood, then peak in number during adolescence and early adulthood and thus becoming stable until the 6th decade^69–71^. After that, melanocytic naevi may fade and disappear or, on the contrary, other pigmented lesions increase in number, especially in males after the 7th decade of life^72–74^, another aspect suggesting caution for the use of nevi in the identification process.

Lastly, we wish to call the attention on other descriptors too and invite researchers to systematically verify their frequencies and uniqueness with a similar approach. We are now working on new systematic research on the frequencies of facial scars whose compound probabilities might be combined with those of facial nevi, strengthening the value that such descriptors might potentially express in the identification of cadavers in specific contexts.

Noticeably, as the results of our study are only preliminary at this phase, since we are still at the first theoretical step clearly based on ideal conditions, any practical approach based on these descriptors in real contexts should be avoided, at least until further studies will be demonstrating that concerns and limitations clearly stated above are negligible in practical settings.

## Conclusions

All in all, this systematic study gained robust preliminary findings that paved the theoretical foundations on the possible use of facial descriptors, facial nevi in our case, for identification purposes. We hope that in the future research focusing on the limitations possibly deriving from real practical settings, as for instance imaging conditions, will be conducted in order to validate this kind of approach in real scenarios. Also, similar systematic researches focused on other facial and body descriptors are desirable in future. Being able to quantify the strength of evidence in identification, regardless of the kind of descriptor or data compared for this purpose, is of paramount importance. Providing such solid probabilistic basis to physical and anthropological descriptors and identifiers is crucial and is becoming more and more a necessity for advancing our discipline. This objective passes through the development of competitive approaches that are as good as genetic methods or those of others primary identifiers in the eyes of the administrators of justice who, in many countries, need to motivate in their sentences the solidity of the science behind the decisions they have made.

Of course, the authors are not suggesting using physical descriptors instead of primary identifier-based methods, but rather to further their use in those contexts where the latter can not be applicable. And, the authors have no doubts that facial nevi, as identifying descriptors, are worthy and potentially useful in the personal identification of victims in many humanitarian and domestic contexts but they still suggest caution in the application of their use in real scenarios, at least until further studies will be examining their effective validity in practical frameworks.

## Data Availability

Due to privacy reasons, data supporting reported results are not publicly available. Requests can be addressed to the corresponding author.
